# Pediatrics

**Published:** 1906-03

**Authors:** Maud J. Frye

**Affiliations:** Buffalo, N. Y.


### Pediatrics.

Conducted by MAUD J. FRYE, M. D., Buffalo, N. Y.

### CAUSES OF INCONTINENCE OF URINE.

CLEMENS (*Archives of Pediatrics,* March 1905), states that in his experience the commonest causes of enuresis and incontinence are:

(1) Hyperacidity of urine. (A condition existing by day and night).

(2) Adenoids. (Nocturnal incontinence—true enuresis).

(3) Chronic interstitial nephritis. (Nocturnal and diurnal incontinence).

(4) Alkalinity of urine. (Due to fixed alkalies—incontinence by day and night).

He uses belladonna but little in treatment. All cases can be treated successfully except those due to organic disease and some cases of hyperacidity and alkalinity. In treating the cases due to alkalinity he uses the acid.

### ENURESIS

Barnes in *American Medicine,* discusses the subject of incontinence of urine in children and states that the successful treatment depends on determining the cause. Enuresis is a symptom of one or more disorders varying from preverted disposition to genuine nervous disease. The author gives a classification of the causes, both organic and functional, of this condition. Every organ and reflex must be carefully studied. The urine and feces must be repeatedly examined. These children must be kept under observation with diligence and without ceasing. The most important elements in the treatment of this condition are diet, hygiene, habit and training.

### DIET.

A generous diet of easily digested food agrees with most children; they should never be overfed. Sometimes a polyuria is associated with an excessive starchy diet, and antidiabetic treatment relieves this condition. In other cases heavy meats and fish have to be eliminated. Stimulating foods and drinks, and liquids of all kinds, should be omitted as much as possible before retiring. In some instances the urine is irritating becaue of its concentration; in these limiting the liquids would aggravate the enuresis. More frequently the urine is abundant, of low specific gravity, and of almost neutral reaction. Many of these children have chronic intestinal indigestion and must be treated accordingly. Emptying the bowel before retiring is a good practice.

### HYGIENE AND HABIT.

Mental and physical irritation should be reduced to a minimum. A quiet, salubrious country place is desirable. Regular habits are imperative. This applies not only to diet, exercise and sleep, but to evacuation of the bowel and bladder. Children should be taught to hold the urine for a considerable time during the day, and should be awakened and made to urinate at a regular hour each night. Exercising in the open air and sunshine is always beneficial. Special exercises directed to strengthening the local muscles can be undertaken with older children. The bath is another hygienic measure often misused. Warm baths are relaxing and frequently followed by colds. The gradually cooling shower or tub bath followed by brisk rubbing, develops vim, vigor and virility. The cold spinal douche gives pleasant results in some instances. A hard bed and moderate covering should always be instituted. Elevation of the foot of the bed and appliances attached to the child to prevent lying on the back are rational measures.

MEDICINAL TREATMENT.

Medical treatment alone is usually negative, unless the enuresis is due to highly acid or irritating urine or infection and inflammation of the urinary mucosa. Then lithium salicylates, potassium citrates and similar drugs give prompt relief. For the general atonic conditions and weakness of the spincters, strychnin, quinin and ergot are of first value. Iron and arsenic should be added when needed. Atropin is the drug commonly prescribed. It influences the muscular and reflex activity by lessening the contractile power of the bladder and diminishing the hyperesthesia of the spinal centers. When depending on this drug it must be given, like quinin for malaria; to obtain effects gradually increasing doses should be continued until the enuresis is controlled or the full action of the drug is obtained. The atropin treatment must be pushed for weeks or months, and when a cure is established the dose may be gradually lessened and the drug slowly withdrawn. When masturbation is practised gelsemium to full physiologic effects may be given alone or with sodium bromid.—*Jour. Am. Medical Association.*

---

Child Life Insurance.—A law has recently been passed in France which prohibits the insurance of children's lives under twelve years of age, an enactment which could be added with advantage to our statutes. The present custom amongst the poor of insuring their children's lives is certainly not for the benefit of the insured, and it may in some instances prove a strong temptation to the perpetuation of infanticide by callous parents and guardians. An infant can be brought to the verge of starvation, and can be slowly done to death by the administration of improper food not in itself unwholesome, and it is often difficult to decide, in such a case, whether the child's miserable condition is owing to lamentable parental ignorance or is the outcome of criminal intention. Marasmic children, whose miserable state has been brought about by improper feeding, are frequently to be found amongst those who have had their lives insured by the weekly payments of trifling sums of money.

The present custom can be defended only on the grounds that it makes provision for the child's funeral expenses, but such a contingency could be better met by providing for the payment of the charges incurred for the interment by the Insurance Company.

Apart from this aspect of the case there is no necessity for the life insurance of a child. The child has no one dependent upon it, no one loses by its death, and there is no monetary value attached to its life.

The passing of this new French law was the outcome of the cruelties practised on insured children in the northern parts of France.—*British Journal of Children's Diseases.*

---

DOSE OF ANTITOXIN IN DIPTHERIA. Fischer *Dietetic and Hygienic Gazette,* January, '05) states that the proper dose of antitoxin for a mild case of diphtheria should never be less than 2,500 to 5,000 units for a child of any age. For a severe case of diptheria with marked toxic symptoms such as a slowness of the pulse and enlarged glands, the dose of antitoxin at the beginning should be between 5,000 and 10,000 units. When a case of diphtheria has been overlooked, and *is seen after the third day of illness* then 10,000 units should be injected. If the symptoms do not improve during the first twenty-four hours, then 5,000 units should be injected after the first twenty-four hours, and every twenty-four hours until there is a marked improvement in the general condition. If a severe case of *septic diphtheria* is seen after the fourth or fifth day of illness then 10,000 units should be injected. If the symptoms do not improve after twenty-four hours, another injection of 10,000 units should be given. There is no harm done by giving 20,000 units during the first twenty-four hours if a severe case of diphtheria of the nose or a severe case of laryngeal diphtheria is seen. Just as enough calomel or belladonna is necessary to produce the proper systemic effect, likewise antitoxin must be given in proper quantities to neutralise the toxin in the system. If we do not neutralise the toxin in the system by giving the proper antidote, then we must not be surprised to find heart complications (myocarditis), a toxic nephritis or paralysis as a sequela to this disiease.

